# A Rapid Active–Latent–Relapse Murine Model of Tuberculosis Based Blood Transcriptional Signature That Distinguishes Disease Stages

**DOI:** 10.3390/ijms27062554

**Published:** 2026-03-11

**Authors:** Haifeng Li, Junfei Wang, Yu Wang, Fan Liu, Jun Tang, Mengmeng Sun, Lingjun Zhan

**Affiliations:** 1 Institute of Laboratory Animal Science, CAMS & PUMC, Beijing Key Laboratory for Animal Models of Emerging and Remerging Infectious Diseases, Chinese Academy of Medical Sciences and Comparative Medicine Center, Peking Union Medical College, Beijing 100021, China; lihf@cnilas.org (H.L.); s2023007008@pumc.edu.cn (J.W.); 18853858007@163.com (Y.W.); 13521208138@163.com (F.L.); tjun1030@hotmail.com (J.T.); 13011825389@163.com (M.S.); 2 National Center of Technology Innovation for Animal Model, Beijing 100021, China; 3 National Human Diseases Animal Model Resource Center, Beijing 100021, China; 4 State Key Laboratory of Respiratory Health and Multimorbidity, Beijing 100021, China; 5 Key Laboratory of Pathogen Infection Prevention and Control (Peking Union Medical College), Ministry of Education, Beijing 100021, China; 6 NHC Key Laboratory of Comparative Medicine, Beijing 100021, China

**Keywords:** *Mycobacterium tuberculosis*, active–latent–relapse murine tuberculosis model, lncRNA sequencing, transcriptional signature

## Abstract

The lack of reliable diagnostic tools and relapse monitoring for latent tuberculosis infection (LTBI) constitutes a major obstacle to global tuberculosis (TB) control. This highlights an urgent need for robust animal models and predictive biomarkers. To address this, we report the successful establishment of a rapid murine model of recapitulating the active, latent, and relapse phases of TB within a compressed ten-week timeframe—hence termed the rapid multi-stage TB murine model. In this model, mice were first intravenously infected with *Mycobacterium tuberculosis*, followed by a four-week isoniazid (INH) regimen starting at two weeks post-infection. By week six, pulmonary bacterial loads in most mice dropped below the detection limit, signifying the establishment of latency. Reactivation was subsequently triggered by a four-week administration of anti-TNF-α (Tumor Necrosis Factor-α) monoclonal antibody. Leveraging this reproducible and time-efficient model, we performed transcriptomic profiling of peripheral blood and identified a distinct sixteen-gene signature (including *Ets2*, *Fam111a*, *Fosl2*, *Gadd45b*, *Nfkbid*, *Rgs1*, *Bhlhe40*, *Il1r2*, *Clec2d*, *Kmo*, *Lynx1*, *Papd4*, *Trim34a*, *Wrb*, *Nlrp12*, *Spns1*) that dynamically tracks disease progression. Collectively, these findings not only provide a valuable and efficient preclinical tool but also deliver transformable candidate biomarkers with immediate potential to guide the development of novel diagnostic strategies for LTBI surveillance and management.

## 1. Introduction

Tuberculosis (TB), caused by the intracellular pathogen *Mycobacterium tuberculosis* (*Mtb*), remains a formidable global health challenge. Due to its highly adaptive nature, *Mtb* can establish long-term persistence within the host—a state defined as latent TB infection (LTBI) [1]. The World Health Organization (WHO) estimates that approximately one-quarter of the global population has LTBI, creating a vast reservoir from which millions of new active cases arise each year [2,3]. While roughly 90% of infected individuals successfully contain the pathogen, approximately 10% eventually progress to active TB, a risk that is markedly amplified in immunocompromised hosts [2]. Consequently, precise diagnosis of LTBI and predictive monitoring of reactivation risk are pivotal for interrupting transmission chains and curbing the epidemic. Indeed, these capabilities form the cornerstone of the WHO’s “End TB Strategy,” which targets the elimination of TB as a global public health threat by 2035 [3,4].

Despite global efforts, critical bottlenecks persisted in elucidating the mechanisms governing LTBI and reactivation. Major impediments included the lack of reliable molecular biomarkers for early LTBI identification and relapse prediction, as well as the absence of specific therapeutic or prophylactic interventions [5,6,7,8]. These gaps stemmed largely from the inability of current animal models to faithfully recapitulate the complex, multi-stage progression of human TB.

Currently, the animal models most widely used to study TB progression, latency, and reactivation include the low-dose aerosol infection model, the Cornell chemotherapy-induced latency model, and relapse models based on immunodeficiency or immunosuppression. While these approaches partially capture the long-term persistence of *Mycobacterium tuberculosis* in the host, each has important limitations. The low-dose aerosol model infects mice with a small number of bacilli (typically 5–10 CFU) to mimic natural exposure and immune containment; however, bacterial burdens often remain relatively high (approximately 3–4 log_10_ CFU), which does not fully reflect the extremely low bacillary load characteristic of human LTBI. Traditional models such as the Cornell model rely on antimicrobial suppression of acute infection to induce a culture-negative, latent-like state [9,10,11]. Nevertheless, this approach lacks standardized protocols, with substantial variability in key parameters—including inoculum size, drug regimens, and rest periods—across studies. As a result, reproducibility and comparability are limited, and the latent state is frequently unstable, characterized by spontaneous reactivation, difficulty in controlled relapse induction, and potential alterations in bacterial phenotype. Moreover, commonly used relapse inducers, such as glucocorticoids, fail to fully reproduce the natural course or typical clinical manifestations of TB and may introduce confounding effects due to excessive tissue toxicity or nonspecific immunosuppression [12]. Therefore, systematic evaluation and refinement of existing animal models are essential to develop more reliable and biologically relevant experimental platforms. In this context, the present study compares and optimizes different variants of the Cornell-based model to shorten experimental timelines, enhance stability and controllability, and provide a robust framework for investigating the immunological mechanisms underlying latent TB and its reactivation.

Therefore, through systematic optimization of infection parameters, antibiotic treatment regimens, and recurrence-induction strategies, we aim to establish a stable and controllable short-term latent–relapse model. Given the critical role of TNF-α in maintaining the structural integrity of tuberculosis granulomas, and the well-documented association between anti–TNF-α therapy and tuberculosis exacerbation or recurrence, glucocorticoids were replaced with an anti–TNF-α monoclonal antibody regimen to trigger disease reactivation [13,14]. As a result, this model provides improved phenotypic fidelity and greater mechanistic insight compared with conventional relapse-induction protocols. Based on this rationale, we successfully established a rapid, multistage murine model of tuberculosis.

## 2. Results

### 2.1. Establishment and Validation of a Rapid Multi-Stage Murine Model of Tuberculosis

**Figure 1:**
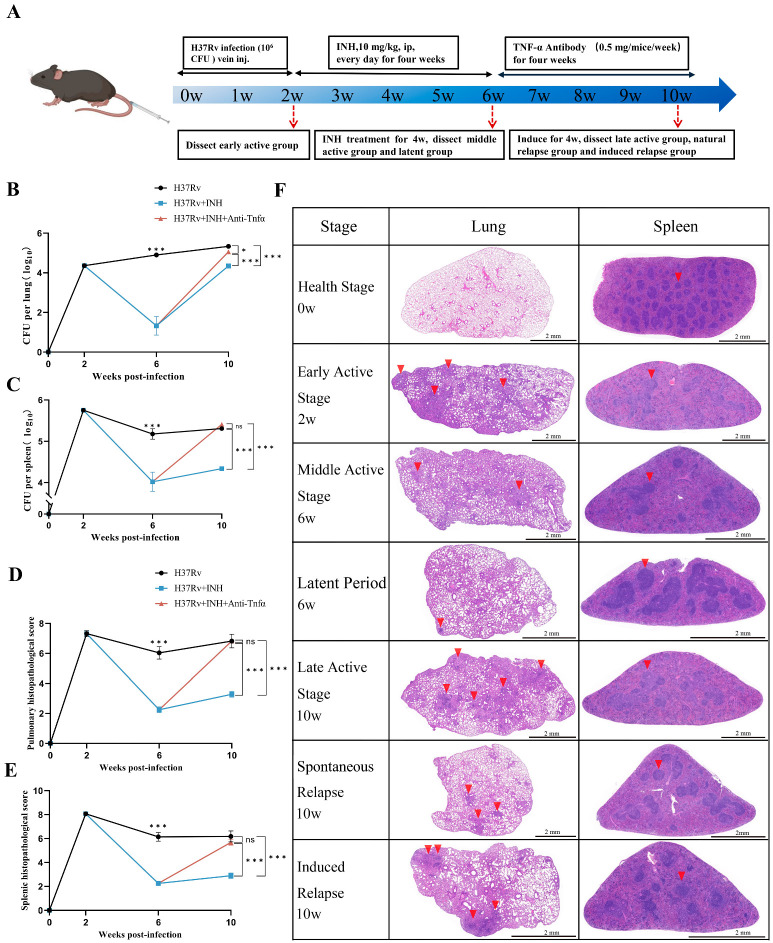
Establishment of an active–latent–relapse-stage murine model of tuberculosis. (**A**) Experimental design and timeline of the active–latent–relapse TB mouse model. Each C57BL/6 mouse was infected intravenously with *Mtb* H37Rv (1 × 10^6^ CFU) and subjected to drug interventions and sample collection at designated time points. (**B**,**C**) Dynamic changes in bacterial loads (CFU) in lung and spleen tissues across different disease stages. (**D**,**E**) Pathological scoring of lung and spleen tissues at various infection stages. (**F**) Representative hematoxylin–eosin (H&E)-stained images of lung and spleen sections from different stages. In the lung sections, red arrows indicate lesion areas. In the spleen sections, red arrows indicate the white pulp regions composed mainly of lymphocytes. Data are shown as mean ± SEM; ns: not significant, *p* > 0.05, * *p* < 0.05, *** *p* < 0.001; *n* = 7.

Through iterative optimization of the modeling conditions, a murine TB model encompassing multiple disease stages was successfully established. Ultimately, a novel modeling workflow was defined that enables the discrimination of three distinct stages of *Mtb* infection within a ten-week timeframe (Figure 1A). Initially, mice were infected via tail vein injection with 1 × 10^6^ CFU of *Mtb*, resulting in established infection over the subsequent two weeks. Starting from Week 3, high-dose INH was administered intraperitoneally daily for four consecutive weeks to suppress bacterial load and induce latency. Beginning at Week 7, reactivation was induced via weekly intraperitoneal injections of anti-TNF-α monoclonal antibodies for four weeks, while a control group received saline. Lung and spleen tissues were harvested at the end of Weeks 2, 6, and 10 for bacterial culture and histopathological analysis (Figure 1A).

Bacterial culture results indicated that at two weeks post-infection, the splenic *Mtb* burden was significantly higher than that of the lungs, exhibiting characteristic features of intravenous infection [15] (Figure 1B,C). By the end of Week 6, pulmonary *Mtb* colonization continued to escalate in the active TB group. Conversely, the INH-treated group showed a precipitous decline in pulmonary bacterial load; specifically, the load in three mice fell below the limit of detection (LOD), while in four others, it was reduced by over 250-fold compared to the concurrent active infection group (Figure 1B and Table 1). A parallel reduction was observed in the spleen (Figure 1C, Supplementary Table S2). At the end of Week 10, pulmonary bacterial burdens in the antibody-treated group rebounded significantly, showing a marked difference from the saline group and approaching levels seen in the concurrent active TB group (Figure 1B). Thus, tissue bacterial burdens confirmed the model’s ability to distinguish between different infection stages.

Histopathological analysis revealed severe immune cell infiltration in the lungs of the active TB group at Weeks 2 and 6. By Week 10, extensive granulomatous regions had formed in the lungs of the active group (Figure 1C,D). In contrast, the area of immune cell infiltration was significantly reduced in the INH-treated group. Following antibody-induced reactivation, tissue pathology in the antibody-treated group was significantly more severe than in the saline control group, further validating the distinct infection phases (Figure 1C,D). Integrating bacterial culture and pathological data, the model yielded samples representing distinct disease states: Early Active TB, Middle Active TB, Late Active TB, Latent TB, Spontaneous Relapse TB, and Induced Relapse TB.

In summary, a simple, rapid, and stable multi-stage murine TB model was successfully established. This model closely mimics the diverse disease progression seen in human *Mtb* infection, effectively recapitulating the “Active–Latent–Relapse” spectrum within a ten-week window.

### 2.2. Transcriptomic Profiling and Identification of Stage-Dependent Gene Signatures

**Figure 2:**
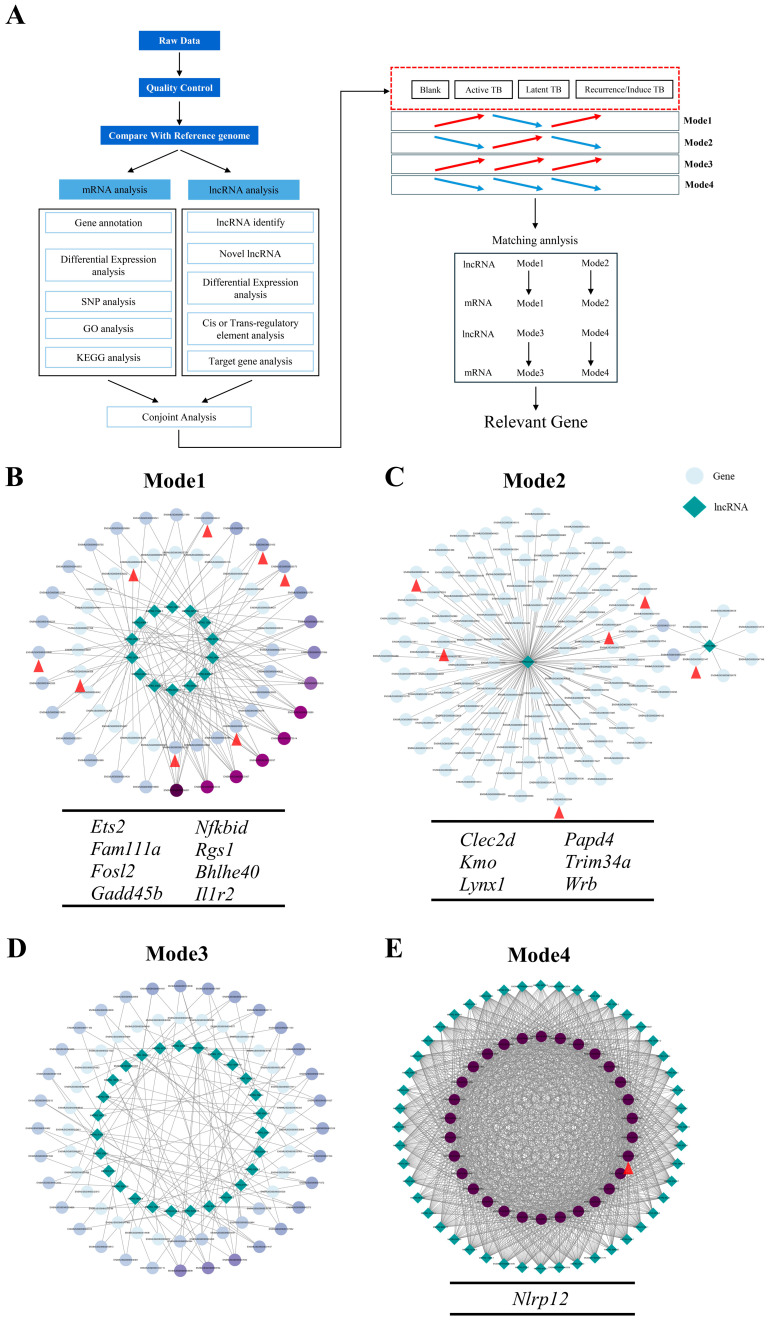
Identification of genes with stable expression changes during distinct infection stages in the murine TB model. (**A**) Workflow for transcriptomic analysis of peripheral blood. After quality control and genome alignment, lncRNA and mRNA analyses were performed, including differential expression, SNP detection, GO and KEGG enrichment, cis/trans regulatory prediction, and target gene identification. Based on the expression dynamics across healthy, active, latent, and relapse stages, gene expression trends were categorized into four patterns (Mode 1–Mode 4), enabling the identification of genes with consistent stage-dependent expression. (**B**–**E**) Co-expression networks between lncRNAs and mRNAs under four expression modes. Each node represents a transcript (lncRNA or mRNA), and edges indicate significant co-expression correlations. The gene indicated by the red arrow is the selected gene. Different modes correspond to distinct temporal expression trends: Mode 1 (**B**) and Mode 2 (**C**) represent stage-specific variations, whereas Mode 3 (**D**) and Mode 4 (**E**) reflect progressive up- or downregulation. Integrative analysis of these modes identified key regulatory genes in peripheral blood during multi-stage TB progression. The red arrows in panels (**B**,**C**,**E**) indicate the genes identified by the screening.

To screen for genes associated with TB progression, lncRNA sequencing was performed on peripheral blood samples from the established multi-stage animal model, covering healthy controls, early active infection, latent infection, induced relapse, and spontaneous relapse groups. Following rigorous quality control and normalization, differential expression analysis identified significantly altered lncRNAs and mRNAs (|log2FC| ≥ 1, *p* < 0.05) (Figure 2A). Subsequently, a two-step screening strategy was implemented to define “associated genes.” First, transcripts were categorized into four longitudinal expression patterns: Mode 1 (up-down-up), Mode 2 (down-up-down), Mode 3 (monotonic increase), and Mode 4 (monotonic decrease) (Figure 2A). Second, a pairing analysis was conducted to identify mRNAs potentially regulated by co-varying lncRNAs within each mode. Potential regulatory links were determined by integrating expression correlation data (Pearson |r| > 0.8, *p* < 0.05) with interaction evidence from databases such as RNAInter and starBase. Consequently, mRNAs meeting both the expression pattern criteria and the lncRNA-pairing requirements were defined as “Relevant Gene”.

Following the initial two-step standard screening, a substantial number of candidate “Relevant Gene” were identified across all expression modes. To prioritize genes with high specificity to TB progression and minimize confounding effects from other pathological conditions, a further refinement step involving a comprehensive literature review was conducted. This process yielded a precise set of candidates: eight genes in Mode 1 (*Ets2*, *Fam111a*, *Fosl2*, *Gadd45b*, *Nfkbid*, *Rgs1*, *Bhlhe40*, and *Il1r2*) (Figure 2B); six genes in Mode 2 (*Clec2d*, *Kmo*, *Lynx1*, *Papd4*, *Trim34a*, and *Wrb*) (Figure 2C); no genes in Mode 3(Figure 2D); and one gene (*Nlrp12*) in Mode 4 (Figure 2E).

Notably, the final gene panel was expanded to include *Spns1* based on its mechanistic relevance. Although the observed expression changes of *Spns1* fell slightly below the strict fold-change threshold, its established role as a key regulator of autophagy, senescence, and mitochondrial metabolism warrants its inclusion in the context of *Mtb* persistence [16,17]. Consequently, a final signature of 16 genes—comprising *Ets2*, *Fam111a*, *Fosl2*, *Gadd45b*, *Nfkbid*, *Rgs1*, *Bhlhe40*, *Il1r2*, *Clec2d*, *Kmo*, *Lynx1*, *Papd4*, *Trim34a*, *Wrb*, *Nlrp12*, and *Spns1*—was established, providing a robust molecular framework for exploring host responses across the multiple stages of TB.

### 2.3. Validation of Stage-Specific Gene Signatures

To validate the authenticity of the identified gene signature, expression profiles of the sixteen candidate genes were assessed in peripheral blood samples from the multi-stage murine TB model. Based on their dynamic expression trajectories across the three distinct disease stages (active-latent-relapse TB), these genes were categorized into four distinct expression patterns.

**Figure 3:**
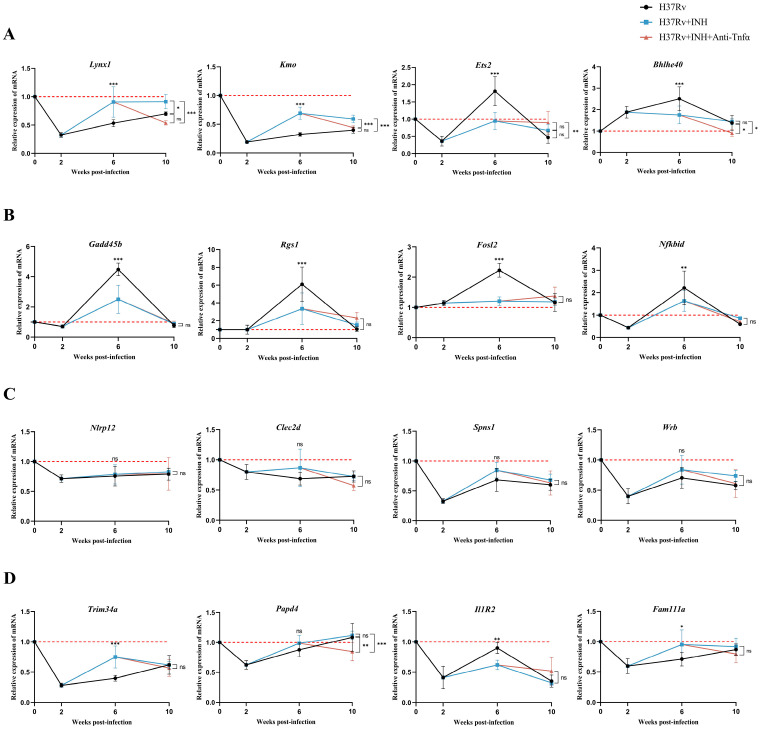
Validation of candidate gene expression in the multi-stage TB mouse model. (**A**–**D**) Relative mRNA expression of sixteen selected genes in peripheral blood samples across infection and treatment stages. Black lines: infection group (*H37Rv* only); blue lines: INH-treated group (*H37Rv* + INH); red lines: anti-TNF-α antibody-induced relapse group (*H37Rv* + INH + anti-TNFα). (**A**) *Lynx1*, *Kmo*, *Ets2*, and *Bhlhe40* were downregulated in the early active stage, returned to near-baseline levels during latency, and declined again during relapse. (**B**) *Gadd45b*, *Nfkbid*, *Fosl2*, and *Rgs1* were upregulated during the mid-active phase and decreased during latency. (**C**) *Nlrp12*, *Clec2d*, *Spns1*, and *Wrb* showed minimal expression changes throughout infection. (**D**) *Trim34a*, *Il1r2*, *Fam111a*, and *Papd4* decreased during active stages, recovered during latency, and declined again upon relapse. The red dashed lines represent the expression levels of the target genes in the healthy control group. Data are presented as mean ± SEM; * *p* < 0.05, ** *p* < 0.01, *** *p* < 0.001, ns: not significant; *n* = 5.

The expression dynamics of *Lynx1*, *Kmo*, *Ets2*, and *Bhlhe40* were closely correlated with disease progression. Two weeks post-*Mtb* infection, peripheral blood levels of *Lynx1*, *Kmo*, and *Ets2* decreased significantly (*p* < 0.05), whereas *Bhlhe40* was markedly upregulated (*p* < 0.001). Following four weeks of high-dose INH treatment, expression levels of all four genes in the latent infection group differed significantly from those in the middle active TB group (*p* < 0.05), and the expression levels of these four genes in the latent infection group at the 6th week tended toward the baseline of the healthy control group (Figure 3A). Upon continuous TNF-α induction for four weeks, levels of *Lynx1* and *Kmo* in the induced relapse group declined again, approaching levels seen in the late active TB group, while *Ets2* and *Bhlhe40* expression gradually recovered (Figure 3A). These results suggest that these four genes may collectively participate in the dynamic regulation of host immune responses throughout the infection–latency–relapse spectrum.

*Gadd45b*, *Rgs1*, *Fosl2*, and *Nfkbid* were significantly upregulated in the middle active TB group. Two weeks post-infection, *Gadd45b* and *Nfkbid* were significantly downregulated, while *Fosl2* and *Rgs1* showed no significant changes. By the middle active infection stage, expression levels of all four genes had risen significantly, suggesting a potential role in immune activation during this phase. In the latent infection group, although levels of *Gadd45b*, *Rgs1*, *Fosl2*, *and Nfkbid* remained higher than in healthy controls, they were significantly lower than in the middle active group, indicating a gradual resolution of the immune response. Finally, at the late active, induced relapse, and spontaneous relapse stages, the expression levels of these four genes tended to approach those observed in the healthy control group (Figure 3B).

Within the multi-stage model, neither INH treatment nor TNF-α-induced relapse altered the expression trends of *Nlrp12*, *Clec2d*, *Spns1*, and *Wrb*, which exhibited high stability. This persistent downregulation suggests an association with immune suppression or the maintenance of host homeostasis during TB infection (Figure 3C).

*Trim34a*, *Il1r2*, *Fam111a*, and *Papd4* displayed reversible expression changes across disease stages (Figure 3D). *Trim34a*, *Papd4*, and *Fam111a* expression dropped rapidly in the early infection phase before gradually recovering. *Il1r2* also decreased rapidly early on but only recovered during the middle active TB stage before declining again.

Collectively, the gene expression curves were consistent with TB disease progression and were validated in the peripheral blood of the multi-stage murine model.

### 2.4. Multigene Expression Profiling Enabled Fine Stratification of Tuberculosis Disease Progression

**Figure 4:**
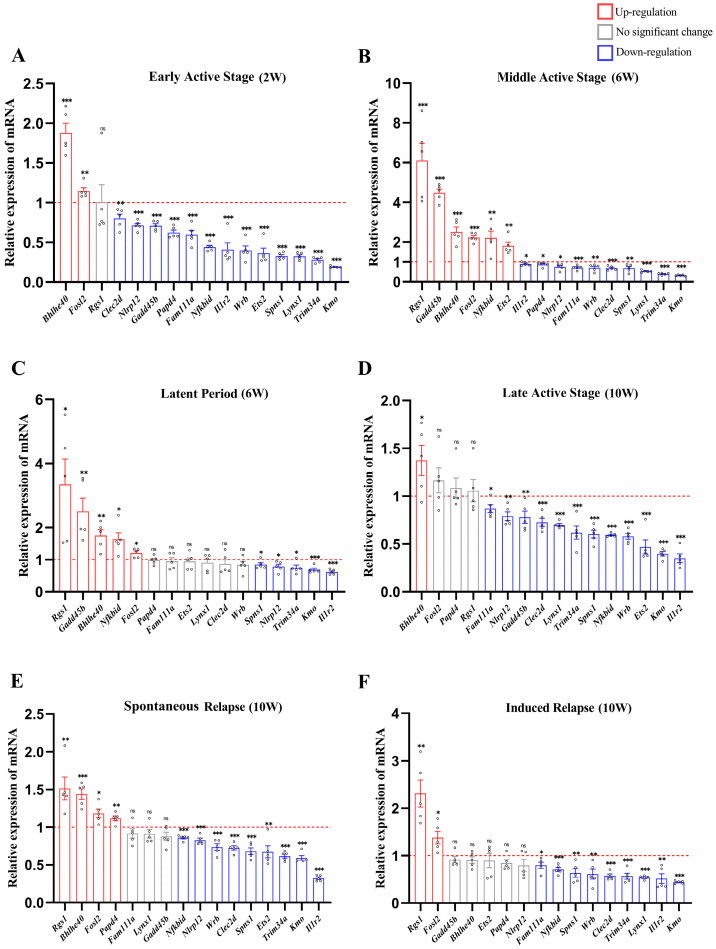
Multigene expression profiling enables refined stratification of tuberculosis progression. (**A**–**F**) Dynamic expression patterns of sixteen candidate genes in peripheral blood across six disease sub-stages following *Mtb* infection: (**A**) early active, (**B**) mid-active, (**C**) latent, (**D**) late active, (**E**) drug-induced relapse, and (**F**) spontaneous relapse. qPCR-validated genes include immune-regulatory, metabolic, and signaling-related molecules that exhibit stage-specific up- or downregulation, enabling discrimination among infection states. The red dashed line represents the baseline expression level of healthy controls. Data are shown as mean ± SEM; * *p* < 0.05, ** *p* < 0.01, *** *p* < 0.001, ns: not significant; *n* = 5.

By delineating the transcriptional dynamics of sixteen genes in peripheral blood across different stages of a multi-stage TB mouse model, the distinct phases of disease progression were effectively stratified. In the Early Active TB group, expression of *Bhlhe40* and *Fosl2* increased, while Rgs1 remained unchanged. Conversely, a broad downregulation was observed for *Clec2d*, *Nlrp12*, *Gadd45b*, *Papd4*, *Fam111a*, *Nfkbid*, *Il1r2*, *Wrb*, *Ets2*, *Spns1*, *Lynx1*, *Trim34a*, and *Kmo*. Overall, the majority of genes in the profile were significantly downregulated compared to the healthy control group (Figure 4A). In the Middle Active TB group, gene expression further polarized. *Rgs1*, *Gadd45b*, *Bhlhe40*, *Fosl2*, *Nfkbid*, and *Ets2* were upregulated, whereas *Il1r2*, *Papd4*, *Nlrp12*, *Fam111a*, *Wrb*, *Clec2d*, *Spns1*, *Lynx1*, *Trim34a*, and *Kmo* were downregulated (Figure 4B). In the Latent TB group, *Rgs1*, *Gadd45b*, *Bhlhe40*, *Nfkbid*, and *Fosl2* remained elevated. *Papd4*, *Fam111a*, *Ets2*, *Lynx1*, *Clec2d*, and *Wrb* showed no significant change, while *Spns1*, *Nlrp12*, *Trim34a*, *Kmo*, and *Il1r2* were downregulated. Notably, expression levels of multiple genes recovered to or approached those of the healthy group, suggesting a relative equilibrium between the host and *Mtb* (Figure 4C). In the Late Active TB group, *Bhlhe40* was upregulated, and *Fosl2*, *Papd4*, and *Rgs1* remained unchanged. However, *Fam111a*, *Nlrp12*, *Gadd45b*, *Clec2d*, *Lynx1*, *Trim34a*, *Spns1*, *Nfkbid*, *Wrb*, *Ets2*, *Kmo*, and *Il1r2* were downregulated. Compared to the Middle Active stage, the expression levels of numerous genes within the signature once again fell below those of the healthy group. Distinct relapse modalities significantly impacted the expression profiles (Figure 4D). In the Induced Relapse group, *Bhlhe40*, *Papd4*, *Ets2*, and *Nlrp12* remained unchanged, while *Fam111a* and *Lynx1* were downregulated (Figure 4E). In contrast, the Spontaneous Relapse group exhibited upregulation of *Bhlhe40* and *Papd4*, downregulation of *Ets2* and *Nlrp12*, and unchanged expression of *Fam111a* and *Lynx1*(Figure 4F). These discrepancies imply underlying mechanistic differences between induced and spontaneous reactivation.

In summary, a systematic analysis of the 16 candidate genes in the peripheral blood of the multi-stage murine TB model revealed dynamic characteristics of the gene expression profile (Table 2).

## 3. Discussion

Historically, LTBI animal models were obtained by three main paradigms: optimization of infection and drug treatment conditions, host gene modification, and the use of attenuated bacteria [18,19,20,21,22]. Building upon the optimization of infection and drug treatment conditions, a rapid multi-stage mouse model exhibiting characteristic pathological and bacteriological features was established and refined, demonstrating strong practical applicability. Peripheral blood from mice at different stages of a multi-stage tuberculosis model was subjected to lncRNA sequencing. Analysis of the dynamic expression patterns of lncRNAs and mRNAs across disease progression revealed a dynamic and multilayered host response network. Key molecular indicators corresponding to each stage of the multi-stage tuberculosis model were subsequently identified. *Kmo*, *Il1r2*, *Trim34a*, and *Spns1*—across all active infection phases form a stable molecular “infection footprint,” effectively distinguishing infected from non-infected states. Furthermore, rapid alterations in *Wrb*, *Spns1*, and *Papd4* marked early active *Mtb* infection, whereas the delayed responses of *Rgs1*, *Gadd45b*, *Fosl2*, and *Nfkbid* represented the peak of immune activation during the middle active infection stage. Collectively, these genes delineate the immune landscape of the multi-stage murine TB model, capturing the critical transitions from acute infection to latency and subsequent relapse.

From a clinical translational perspective, the multigene panel identified in this study demonstrates clear application potential. The “persistently low expression” signature composed of *Kmo*, *Il1r2*, *Trim34a*, and *Spns1*—due to its high consistency—mitigates the risk of confounding by single treatments (e.g., medication) or short-term immune fluctuations, making it a promising candidate panel for infection screening. To advance clinical application, subsequent validation of transcript and protein levels in independent human cohorts is warranted. This includes evaluating the robustness and sensitivity of these candidate genes across diverse backgrounds and verifying their diagnostic and predictive performance through ROC analysis, cross-validation, and multi-center studies.

When compared with prior studies, it appeared that several candidate genes were also reported in human TB. For instance, *Bhlhe40* transcriptional downregulation in active TB patients presents a pattern divergent from the upregulation observed in the murine model [23]. This discrepancy may be attributable to distinct disease stages or interspecies variations in host status. Nonetheless, both models converge on *Bhlhe40* as a pivotal immunoregulatory factor in the host response to *Mtb*. Additionally, *Gadd45b* is recognized as a critical mediator of apoptosis in *Mtb*-specific CD4^+^ T cells during active TB [24]. *RGS1* demonstrates a distinct cell type-dependent duality: correlating negatively with nutritional indices in CD39^+^ Th1 cells, while serving as a discriminatory biomarker for osteoarticular TB (OTB) in macrophages [25,26]. Intriguingly, the anti-inflammatory decoy receptor *IL1R2* is upregulated in active TB patients—a finding contrasting with the murine model—suggesting it may limit excessive IL-1-mediated inflammation, thereby serving as a potential biomarker distinguishing active TB from LTBI [27]. These comparisons underscore the evolutionary conservation of core immune pathways while emphasizing the imperative for cautious transformation validation.

In contrast, the remaining 12 candidates (*Ets2*, *Fam111a*, *Fosl2*, *Nfkbid*, *Clec2d*, *Kmo*, *Lynx1*, *Papd4*, *Trim34a*, *Wrb*, *Nlrp12*, and *Spns1*) have not yet been directly implicated in TB pathogenesis. This underscored the novelty of the findings, broadening the repertoire of potential immune regulatory genes. Future studies should aim to evaluate the concordance of these gene expression profiles in high-burden human populations, complemented by protein-level quantification and functional assays to establish causal relationships and elucidate their precise roles in TB pathogenesis. Given the limitations of the present study, a larger longitudinal cohort will be necessary to formally assess the correlation between gene expression and tuberculosis progression over time.

Several limitations of this study are acknowledged. First, inherent differences between mice and humans in immune composition, granuloma structure, and pathological progression may limit the extrapolation of certain peripheral blood signals. Second, the current analysis is primarily based on transcriptomic data; therefore, validation of the expression levels and cellular origins of key molecules using proteomics and single-cell sequencing is required. Third, functional inferences for several genes are based on existing annotations and require experimental verification to determine their specific roles in TB pathogenesis. Finally, sex is a considerable biological variable in tuberculosis research, since male neonatal mice have been reported to be more susceptible to *Mtb*, potentially due to the immunomodulatory effects of testosterone [28]. In this study, only female mice were used to establish the model in order to maintain consistency with our previous work employing this infection model. Whether the parameters and characteristics of this model differ between female and male mice remains an open question and warrants further experimental comparison. To address these limitations, future investigations should focus on multi-center validation in human cohorts, the establishment of clinical assays centered on protein markers, and gene function intervention experiments to elucidate key pathways. These steps will facilitate the translation of basic findings into actionable diagnostic or interventional strategies.

In summary, the primary contribution of this study is the successful development of an efficient and robust murine model that simulates the active–latent–relapse stages of TB. The model stands out due to two key innovations that enhance its translational relevance. First, it employs a precisely controlled short-term INH regimen to induce a drug-mediated latent state, closely mirroring the clinical reality of post-treatment latency. Second, relapse is triggered through the administration of an anti–TNF-α monoclonal antibody blocker, which accurately reproduces the specific immune perturbation observed in patients receiving such therapy, rather than relying on nonspecific immunosuppressive approaches [29,30]. Based on this multi-stage model, a signature of sixteen genes associated with disease progression was screened, providing a vital model foundation and a set of candidate molecules for investigating and detecting the progression of TB.

## 4. Materials and Methods

### 4.1. Ethics Statement and Biosafety Guarantee

This research was performed in accordance with the Guide for the Care and Use of Laboratory Animals. All procedures were reviewed and approved by the Institutional Animal Care and Use Committee (IACUC) of the Institute of Laboratory Animal Science (ILAS), Chinese Academy of Medical Sciences (CAMS), and the approval No. was ZLJ24005. The biosafety of all experiments involving *Mtb* infection, both in vitro and in vivo, was assessed and approved by the Administration Committee of the Animal Biosafety Level 3 Laboratory (ABSL-3) at ILAS, CAMS.

### 4.2. Strains

*Mtb* strain H37Rv (ATCC27294) was cultured at 37 °C in Middlebrook 7H9 broth (BD, cat 262710) supplemented with 0.05% Tween 80, 0.2% glycerol, and oleic acid-albumin-dextrose-catalase (OADC; BD, cat 212351, Franklin Lakes, NJ, USA). 

### 4.3. Mice Infection

Female 6–8 weeks SPF C57BL/6 mice were purchased from Charles River [SCXK(Jing)2021-0012], [SYXK(Jing)2024-0012], which were maintained under controlled environmental conditions (temperature 23 ± 2 °C, humidity 50 ± 10%, 12/12 h light/dark cycle) with ad libitum access to food and water (five mice per cage). After a 5-day ABSL-3 adaptation period, the mice were infected intravenously via the tail vein with *Mtb* H37Rv at a dose of 1 × 10^6^ colony-forming units (CFU) in 100 μL of phosphate-buffered saline (PBS) per mouse, and the infection dose was confirmed by plating serial dilutions on Middlebrook 7H10 agar. Subsequently, mice received daily intraperitoneal injections of isoniazid (Sigma, cat PHR1932, St. Louis, MO, USA) from the 3rd to 6th week post-infection, while recombinant mouse TNF-α (MCE, cat HY-P99148, Monmouth Junction, NJ, USA) was administered intraperitoneally at 0.5 mg per mouse once weekly from 7th to 10th week post-infection. Finally, mice were euthanized at the scheduled time points, lungs and spleens were aseptically harvested, half of each lung or spleen was homogenized in sterile PBS, and bacterial loads were determined by plating serial dilutions of tissue homogenates on Middlebrook 7H10 agar at 37 °C for 3–4 weeks and counting CFUs after incubation. The other half of the lung and spleen were fixed for histopathologic analysis.

### 4.4. Peripheral Blood Transcriptome lncRNA Sequencing

Peripheral blood samples were collected from mice at the indicated time points post-infection. Total RNA was extracted using the Qiagen RNeasy Mini Kit according to the manufacturer’s instructions. RNA concentration and purity were assessed using a NanoDrop ND-2000 spectrophotometer (Thermo Fisher Scientific, Waltham, MA, USA), and RNA integrity was evaluated with an Agilent 2100 Bioanalyzer (Agilent Technologies, Santa Clara, CA, USA). Only high-quality RNA samples (OD260/280 = 1.8–2.2, OD260/230 ≥ 2.0, RNA integrity number [RIN] ≥ 6.5–7.0, 28S/18S ≥ 1.0, and total RNA ≥ 1 μg) were used for library construction. Strand-specific RNA sequencing libraries were prepared using the NEBNext^®^ Ultra™ RNA Library Prep Kit (New England Biolabs, Ipswich, MA, USA), following the manufacturer’s protocols. Libraries were pooled and sequenced on an Illumina NovaSeq platform (NovaSeq X Plus or NovaSeq 6000, Illumina, San Diego, CA, USA) to generate paired-end reads. Raw sequencing data were processed following a standardized operating procedure. Quality control and adapter trimming were performed using fastp to obtain clean reads. Clean reads were aligned to the mouse reference genome (GRCm38.84 or GRCm39, Mus musculus) using HISAT2. Transcript assembly and quantification were conducted with StringTie, and gene expression levels were calculated as fragments per kilobase of transcript per million mapped reads (FPKM). For lncRNA identification, assembled transcripts were compared with reference annotations and lncRNA databases to identify known lncRNAs, after excluding transcripts overlapping known mRNAs. Candidate lncRNAs were further filtered based on established criteria, including transcript length > 200 bp and exon number ≥ 2. Differential expression analysis was performed using DESeq2, with genes meeting the criteria of |log_2_ fold change| ≥ 1 and adjusted *p*-value (q-value) < 0.05 considered significantly differentially expressed. Differentially expressed lncRNAs were further analyzed, and co-expression analyses (e.g., lncRNA–mRNA networks) were used to predict their potential functional associations.

All sequencing and bioinformatic analyses were conducted in accordance with the standardized lncRNA sequencing and analysis workflow provided by Meiji Biotechnology Co., Ltd. (Shanghai, China).

### 4.5. Total RNA Extraction and RT-qPCR

Peripheral blood samples from mice were collected in anticoagulant tubes, and red blood cells were removed using a red blood cell lysis buffer. The remaining cell fraction was collected for total RNA extraction and purification using the Qiagen RNeasy Mini Kit according to the manufacturer’s instructions. After RNA collection and concentration, 0.4 μg of total RNA from each sample was reverse-transcribed into complementary DNA (cDNA) using the RT Master Mix for qPCR (MCE, cat HY-K0511, Monmouth Junction, NJ, USA). Quantitative real-time PCR (qRT-PCR) was then performed on a QuantStudio™ 3 Real-Time PCR System (Applied Biosystems, Foster City, CA, USA) using SYBR Green qPCR Master Mix (MCE, cat HY-K0501A, Monmouth Junction, NJ, USA). The relative expression levels of target genes were normalized to *Gapdh* and calculated using the 2^−ΔΔCt^ method.

### 4.6. Histopathological Analysis

Lung and spleen tissues were fixed in 4% paraformaldehyde, embedded in paraffin, and sectioned for hematoxylin–eosin (H&E) staining. Histological images were acquired using the VIEW 2 imaging system (Hamamatsu Photonics, Hamamatsu City, Shizuoka, Japan). Histopathological scoring was independently performed by multiple qualified pathologists under blinded conditions. Scores were assigned based on the extent and severity of lesions in the lung and spleen tissues, with normal tissues assigned a score of 0. Increasing values indicated more severe pathological changes, up to a maximum score of 10 [31].

### 4.7. Bioinformatics Analysis of Differentially Expressed lncRNAs and mRNAs

Raw sequencing data underwent stringent quality control to yield high-quality clean reads, which were mapped to the reference genome using HISAT2. Next, transcript assembly and novel lncRNAs identification were executed using StringTie. Thereafter, differential expression analysis of lncRNAs and mRNAs was performed using edgeR, with the data modeled under a negative binomial distribution. Meanwhile, the biological functions of differentially expressed genes and predicted lncRNA targets were elucidated by GO/KEGG enrichment analyses and Goatools/KOBAS, respectively, combined with comprehensive quality checks, including sequencing saturation and sequence consistency analyses. Ultimately, an lncRNA–mRNA co-expression network was established, enabling characterization of expression patterns and systematic screening for candidate target genes that meet specific selection criteria.

### 4.8. Statistical Analysis

Data were analyzed using GraphPad Prism 9.0 (GraphPad Software, Inc., San Diego, CA, USA). Statistical significance was determined by Student’s *t*-test for making comparisons between two groups, and one-way analysis of variance (ANOVA) was employed for making comparisons among three or more groups. *p* < 0.05 was indicative of statistical significance.

## Figures and Tables

**Table 1:** Bacterial load (CFU) in lung tissues of mice during different stages of TB progression.

Stage	Colony-Forming Unit (CFU) in the Lung						
Health Stage (0 W)	0	0	0	0	0	0	0
Early Active Stage (2 W)	34,000	22,000	38,000	30,000	12,200	20,000	20,600
Middle Active Stage (6 W)	80,000	80,000	80,000	80,000	60,000	110,000	80,000
Latent Period (6 W)	0	0	0	200	180	200	280
Late Active Stage (10 W)	180,000	280,000	200,000	200,000	240,000	234,000	200,000
Spontaneous Relapse (10 W)	27,800	21,200	23,400	22,200	25,400	17,800	20,000
Induced Relapse (10 W)	80,000	88,000	114,000	118,000	154,000	98,000	194,000

**Table 2:** Dynamic expression patterns of candidate genes are related to healthy controls during tuberculosis progression.

	Stage	Early Active Stage (2 W)	Middle Active Stage (6 W)	Latent Period (6 W)	Late Active (10 W)	Spontaneous Relapse (10 W)	Induced Relapse (10 W)
Gene							
*Kmo* **^1^**		↓	↓	↓	↓	↓	↓
*Trim34a* **^1^**		↓	↓	↓	↓	↓	↓
*Il1r2* **^2^**		↓	↓	↓	↓	↓	↓
*Spns1* **^1^**		↓	↓	↓	↓	↓	↓
*Fam111a* **^1^**		↓	↓	→	↓	↓	→
*Wrb* **^1^**		↓	↓	→	↓	↓	↓
*Lynx1* **^1^**		↓	↓	→	↓	↓	→
*Clec2d* **^1^**		↓	↓	→	↓	↓	↓
*Gadd45b* **^2^**		↓	↑	↑	↓	→	→
*Nfkbid* **^1^**		↓	↑	↑	↓	↓	↓
*Ets2* **^1^**		↓	↑	→	↓	→	↓
*Nlrp12* **^1^**		↓	↓	↓	↓	→	↓
*Bhlhe40* **^2^**		↑	↑	↑	↑	→	↑
*Rgs1* **^2^**		→	↑	↑	→	↑	↑
*Fosl2* **^1^**		↑	↑	↑	→	↑	↑
*Papd4* **^1^**		↓	↓	→	→	→	↑

Note: Arrows indicate the direction of gene expression change relative to healthy controls: → represents no significant difference in expression; ↑ represents a significant increase (upregulation); ↓ represents a significant decrease (downregulation). Numerical labels indicate whether the gene is reported or not in TB, **^1^**: Not reported in TB. **^2^**: Previously reported or characterized in TB.

## Data Availability

The original contributions presented in this study are included in the article. Further inquiries can be directed to the corresponding author.

## Supplementary Materials

The following supporting information can be downloaded at https://www.mdpi.com/article/10.3390/ijms27062554/s1.

## Abbreviations

The following abbreviations are used in this manuscript:

LTBI	Tuberculosis latent tuberculosis infection
TB	Tuberculosis
INH	Isoniazid
*Mtb*	*Mycobacterium tuberculosis*
WHO	World Health Organization
TNF-α	Tumor Necrosis Factor-α
lncRNA	Long non-coding RNA
mRNA	Messenger RNA
CFU	Colony-Forming Unit
LOD	Limit of Detection
SNP	Single Nucleotide Polymorphism
OTB	Osteoarticular TB
