# Supplementary material for: A Rapid Active–Latent–Relapse Murine Model of Tuberculosis Based Blood Transcriptional Signature That Distinguishes Disease Stages
Source: Int J Mol Sci. 2026 Mar 11;27(6):2554. doi: 10.3390/ijms27062554 (PMC13027359; doi:10.3390/ijms27062554)
Supplement: Supplementary file 1 [file ijms-27-02554-s001.zip › Supplementary File S1 qPCR primer sequence.pdf]

Supplement table S1 qPCR primer sequence

Gene name	Primer(5'to3')
Gapdh-F	GAGTCTACTGGTGTCTTC
Gapdh-R	AATCTTGAGTGAGTTGTC
Clec2d-F	GGTTTGACAACCAGGATGAGC
Clec2d-R	TCTCCCCGGATGGGAATCG
Lynx1-F	ACCCATCTGCTCACAGTGTTC
Lynx1-R	TCGTGTGGTCATACAGTAGGTG
Rgs1-F	TCTGGGATGAAATCGGCCAAG
Rgs1-R	GCATCTGAATGCACAAATGCTT
Spns1-F	CCAAGCAGATGATCCTGATGAC
Spns1-R	TGTAGCCCCTCACAGTCTGG
Il1r2-F	GTTTCTGCTTTCACCACTCCA
Il1r2-R	GAGTCCAATTTACTCCAGGTCAG
Bhlhe40-F	ACGGAGACCTGTCAGGGATG
Bhlhe40-R	GGCAGTTTGTAAGTTTCCTTGC
Ets2-F	CCTGTCGCCAACAGTTTTCG
Ets2-R	TGGAGTGTCTGATCTTCACTGA
Fam111a-F	ATGAGCTGTAAGAAGCGGAAATC
Fam111a-R	GTGTTGGTTATATCCCTTGGCA
Fosl2-F	CCAGCAGAAGTTCCGGGTAG
Fosl2-R	GTAGGGATGTGAGCGTGGATA
Gadd45b-F	CAACGCGGTTCAGAAGATGC
Gadd45b-R	GGTCCACATTCATCAGTTTGGC
Nfkbid-F	TCCCCACAGTTGCCTTCAC
Nfkbid-R	GAGCGGCGTCTTGCCTTTA
Kmo-F	ATGGCATCGTCTGATACTCAGG
Kmo-R	CCCTAGCTTCGTACACATCAACT
Papd4-F	AAACTCAATTTTGGGTCGTCCA
Papd4-R	GTGCATCTATAAGTTGCTGGTGT
Trim34a-F	GTAATAACGGTATCTTGGGCTCC
Trim34a-R	TGCGTTGTCTAACATCAAACCTT
Wrb-F	TCAAAACGCACGTGAAGGC
Wrb-R	TAGTGGGGTGATCCACTTGC
Nlrp12-F	GACGAATGGAGAAGGCTGGT
Nlrp12-R	ACCATTATTTGGAGTGACCCTCA
